# Current Evidence on the Use of Biomarkers for the Diagnosis of Acute Mesenteric Ischemia: A Narrative Review

**DOI:** 10.7759/cureus.112865

**Published:** 2026-07-17

**Authors:** Maria D Velikoudi, Charis G Kourgiali, Kalliopi-Kleio E Gotti, Dimitrios A Chatzelas

**Affiliations:** 1 2nd Department of Surgery, “G. Gennimatas” General Hospital, Faculty of Medicine, Aristotle University of Thessaloniki, Thessaloniki, GRC

**Keywords:** acute mesenteric ischemia, biomarker, diagnosis, intestinal ischemia, laboratory test

## Abstract

Acute mesenteric ischemia (AMI) is a life-threatening vascular emergency associated with high morbidity and mortality, largely because diagnosis is frequently delayed by non-specific clinical manifestations. Although computed tomography angiography (CTA) remains the diagnostic gold standard, considerable research has focused on identifying laboratory biomarkers that may facilitate earlier recognition and improve clinical decision-making. This narrative review summarizes the current evidence regarding routinely available and emerging laboratory biomarkers for the diagnosis of AMI. A comprehensive literature search was performed using PubMed/Medical Literature Analysis and Retrieval System Online (MEDLINE), Scopus, Web of Science, and the Cochrane Library, supplemented by manual review of reference lists. Routine laboratory markers, including leukocyte count, C-reactive protein (CRP), serum lactate, metabolic acidosis, D-dimer, amylase, phosphate, and procalcitonin (PCT), were evaluated alongside investigational biomarkers, such as intestinal fatty acid-binding protein (I-FABP), D-lactate, citrulline, ischemia-modified albumin (IMA), alpha-glutathione S-transferase (α-GST), interleukin-6 (IL-6), and cell-count-derived inflammatory indices. Current evidence demonstrates that no single laboratory biomarker possesses sufficient sensitivity and specificity to independently confirm or exclude AMI. Serum lactate and metabolic acidosis primarily reflect advanced intestinal injury and systemic hypoperfusion, rather than early ischemia, whereas D-dimer offers relatively high sensitivity but poor specificity. Enterocyte-specific biomarkers, particularly I-FABP, remain biologically attractive because they may detect early mucosal injury; however, inconsistent diagnostic performance, limited assay availability, and methodological heterogeneity have prevented routine clinical implementation. Emerging multimarker strategies integrating indicators of enterocyte injury, coagulation activation, inflammation, and tissue hypoperfusion may improve diagnostic accuracy but require prospective validation. At present, laboratory investigations should be regarded as complementary tools that increase clinical suspicion, assist with risk stratification, and reinforce the need for urgent CTA. They should never delay definitive imaging or revascularization when AMI is suspected. Further high-quality prospective studies are required to establish standardized biomarker panels capable of facilitating earlier diagnosis and improving patient outcomes.

## Introduction and background

Acute mesenteric ischemia (AMI) refers to sudden intestinal hypoperfusion resulting from arterial embolism, arterial thrombosis, non-occlusive mesenteric ischemia, or mesenteric venous thrombosis (MVT) [1,2]. Although relatively uncommon compared to other causes of acute abdomen, it carries disproportionately high mortality because irreversible intestinal injury may develop before the diagnosis is established [1-3]. The classic teaching of severe abdominal pain out of proportion to physical findings is important but insensitive, particularly in elderly, sedated, critically ill, or postoperative patients [1,2]. Contemporary guidelines emphasize that early computed tomography angiography (CTA) is the diagnostic modality of choice and should not be postponed while awaiting laboratory confirmation [1,2].

The search for an accurate biomarker is therefore clinically appealing. An ideal laboratory marker should be rapidly available, inexpensive, reproducible, elevated early after mucosal ischemia, specific for intestinal injury, and capable of distinguishing reversible ischemia from transmural necrosis [3]. Despite extensive study, this biomarker has not yet entered routine clinical practice [3-5]. Most available laboratory abnormalities reflect inflammation, hypoperfusion, coagulation activation, bacterial translocation, or established necrosis, rather than early intestinal ischemia itself [3-6]. Consequently, clinicians continue to rely primarily on clinical suspicion and urgent vascular imaging, despite decades of biomarker research.

The objective of this narrative review was to critically evaluate the current evidence regarding laboratory biomarkers for the diagnosis of AMI. Specifically, we summarize the biological rationale, diagnostic performance, and clinical applicability of routinely available laboratory tests and emerging biomarkers, discussing their limitations, and integrating the findings with contemporary guideline recommendations. Finally, we propose a practical framework for interpreting laboratory abnormalities alongside clinical assessment and CTA.

## Review

Methods

A narrative literature review was performed using PubMed/Medical Literature Analysis and Retrieval System Online (MEDLINE), Scopus, Web of Science, and Cochrane electronic databases up to June 2026. Search terms included “acute mesenteric ischemia," “intestinal ischemia," "biomarker," “laboratory marker," "lactate," "D-dimer," “intestinal fatty acid-binding protein," "I-FABP," "D-lactate," "citrulline," “ischemia-modified albumin,” “alpha-glutathione S-transferase," "procalcitonin," “neutrophil-to-lymphocyte ratio," and "diagnosis." The reference lists of relevant articles were screened for additional sources. Only articles published in English and indexed in peer-reviewed journals were included. Priority was given to human studies, systematic reviews, meta-analyses, and recent guidelines, particularly the 2022 World Society of Emergency Surgery guidelines and the 2025 European Society for Vascular Surgery guidelines [1,2]. Experimental studies were included when human evidence was limited and when they clarified biological plausibility.

Pathophysiological basis of biomarker release

The diagnostic difficulty of AMI is partly explained by the layered and time-dependent nature of intestinal ischemic injury. Early ischemia initially affects the mucosa, which is metabolically active and vulnerable to hypoxia. With ongoing hypoperfusion, injury progresses to the submucosa, muscularis, and serosa, eventually causing transmural necrosis, perforation, bacterial translocation, systemic inflammatory response (SIR), and shock [6,7]. Biomarkers released at different stages, therefore, reflect different biological processes. Enterocyte-derived proteins, such as intestinal fatty acid-binding protein (I-FABP), are intended to reflect early mucosal injury, whereas lactate, metabolic acidosis, leukocytosis, and organ dysfunction markers often reflect later systemic consequences. Reperfusion may further augment oxidative stress and inflammatory mediator release, thereby influencing biomarker kinetics [3,6-8].

This time dependency is crucial. A normal lactate early in the course of AMI does not exclude intestinal ischemia, while a high lactate may indicate late disease, extensive necrosis, septic shock, or global hypoperfusion rather than AMI specifically [1,2,9]. Similarly, D-dimer may rise during thrombus formation and fibrinolysis, but is also elevated in infection, malignancy, trauma, venous thromboembolism (VTE), aortic syndromes, recent surgery, and advanced age [10]. Therefore, the clinical value of a biomarker depends not only on its analytical performance but also on timing [3].

Routine laboratory markers

Leukocytosis and C-reactive Protein

Leukocytosis is among the most frequent laboratory abnormalities in AMI, but it is neither sensitive enough to exclude the disease nor specific enough to confirm it [1,2]. White blood cell elevation reflects systemic inflammation, stress response, bowel necrosis, or sepsis and overlaps with appendicitis, diverticulitis, pancreatitis, perforated viscus, bowel obstruction, and many other acute abdominal conditions. C-reactive protein (CRP) has similar limitations. It may be elevated in advanced ischemia or inflammatory complications, but usually lacks the temporal precision required for early diagnosis [3,4].

Cell-count-derived indices, including the neutrophil-to-lymphocyte ratio (NLR), have been evaluated as inexpensive adjuncts. Retrospective studies suggest that NLR may be higher in AMI than in some control populations and may correlate with severity or mortality. However, NLR is fundamentally an inflammatory stress marker and is affected by infection, malignancy, corticosteroid use, trauma, and postoperative status [11,12]. Its current role is therefore supportive rather than diagnostic. A markedly elevated NLR may strengthen suspicion in a compatible clinical scenario, but it should not be used as a screening test or as a reason to delay CTA [1,2].

Lactate and Metabolic Acidosis

Serum L-lactate is widely used in emergency and critical care practice and is often perceived as a key test for AMI. However, its diagnostic role is limited. Lactate rises when anaerobic metabolism, impaired clearance, shock, sepsis, or extensive tissue hypoxia is present. In AMI, this usually occurs after significant bowel injury or systemic compromise, meaning that lactate is more useful as a marker of severity than as an early diagnostic test [1,2,9,13]. Guidelines caution that normal lactate values do not exclude AMI [1,2]. This is especially important in early arterial occlusion, isolated mucosal ischemia, or venous ischemia, before systemic deterioration. Conversely, elevated lactate is non-specific and may be caused by dehydration, sepsis, cardiac failure, hepatic dysfunction, seizures, diabetic ketoacidosis, beta-agonist use, or generalized hypoperfusion. Serial lactate measurement may help monitor deterioration and response to treatment, but diagnosis should be based on clinical suspicion and CTA, rather than lactate thresholds alone [13,14].

Metabolic acidosis, base deficit, low bicarbonate, and elevated anion gap are similarly late and non-specific findings. Their presence in a patient with acute abdominal pain, atrial fibrillation, atherosclerosis, shock, or vasopressor exposure should heighten concern for bowel ischemia, but their absence should not reassure clinicians when the clinical picture is compatible with AMI [13,14].

D-dimer

D-dimer is one of the most studied laboratory markers in AMI. Its biological rationale is strong: arterial embolism, arterial thrombosis, venous thrombosis, and microvascular coagulation can activate fibrinolysis, producing elevated D-dimer levels. Several studies suggest high sensitivity but low specificity for AMI [10,15,16]. A meta-analysis reported a pooled sensitivity of 0.94 but a specificity of only 0.50, indicating that D-dimer may be more useful as a rule-out adjunct in selected low-risk patients, rather than as a confirmatory test [15].

The clinical problem is that many patients at risk for AMI are elderly, hospitalized, septic, postoperative, or have cancer, cardiovascular disease, renal dysfunction, or thromboembolic disease, all of which can elevate D-dimer. Therefore, a positive D-dimer has poor discriminatory value in the population in which AMI is most suspected. A normal D-dimer may reduce the probability of thrombotic or embolic AMI, but current evidence is insufficient to recommend withholding CTA solely because D-dimer is normal [1,2,4,15]. D-dimer may be most useful when incorporated into structured diagnostic pathways with clinical risk assessment, but this strategy requires prospective validation [1,2].

Amylase, Liver Enzymes, Phosphate, and Other Routine Tests

Hyperamylasemia has been reported in AMI and may lead to misdiagnosis as pancreatitis. The mechanism may involve pancreatic hypoperfusion, intestinal injury, or non-specific abdominal inflammation. However, amylase is not sufficiently specific and should not divert attention from AMI when vascular risk factors or disproportionate pain are present [1]. Liver enzymes, bilirubin, creatinine, and coagulation abnormalities are also non-specific but may identify multi-organ dysfunction, shock, dehydration, or comorbidity [4,5].

Serum phosphate has been explored because cellular breakdown and necrosis may release phosphate during advanced ischemia. However, phosphate elevation is inconsistent, may be late, and is influenced by renal function, acid-base status, and tissue injury from many causes [17]. Potassium and pH have also been studied, but their diagnostic utility remains insufficient for routine decision-making [18]. In practice, these tests are best interpreted as severity markers rather than diagnostic markers [1].

Emerging and investigational biomarkers

Intestinal Fatty Acid-Binding Protein

I-FABP is a small cytosolic protein expressed in mature enterocytes, especially at the villus tips, which are highly susceptible to ischemia. Because it is released rapidly after enterocyte injury, I-FABP has been proposed as a marker of early intestinal ischemia [6,8,19-23]. A meta-analysis suggested promising diagnostic performance for AMI, with a pooled sensitivity of around 0.80 and a specificity of around 0.85 [19]. Urinary I-FABP has also been investigated as a non-invasive marker and may have diagnostic potential [22].

However, subsequent evidence has been less conclusive. In a large cross-sectional study from France, plasma I-FABP failed to distinguish AMI from other causes of acute abdominal pain, with poor discriminatory performance [21]. Systematic reviews emphasize heterogeneity in assays, timing, thresholds, AMI definitions, control groups, and disease severity [3,4]. I-FABP remains one of the most biologically plausible biomarkers, but it is not yet ready for routine clinical decision-making [1,2]. Its future may lie in combination panels or serial measurement, rather than isolated testing.

D-lactate

D-lactate is produced by intestinal bacteria and may rise when the mucosal barrier injury permits bacterial translocation or altered intestinal metabolism. It is conceptually attractive because it may reflect intestinal rather than systemic lactate metabolism [6,23]. However, D-lactate assays are not widely available in emergency practice, and published studies showed variable performance. Reviews have concluded that evidence is insufficient to support D-lactate as a standalone diagnostic marker [9,23]. In the large study by Nuzzo et al., D-lactate did not differentiate AMI from other causes of acute abdominal pain [20]. Its current role is investigational.

Citrulline

Citrulline is mainly produced by small-bowel enterocytes and has been used as a marker of functional enterocyte mass. Low citrulline levels may reflect extensive mucosal loss or intestinal failure, making it biologically relevant to ischemic injury [6,20]. However, citrulline is influenced by renal function, chronic intestinal disease, nutritional status, and the extent of viable enterocyte mass. In acute diagnosis, it has not shown sufficient accuracy. Nuzzo et al. found that citrulline was not reliable for differentiating AMI from non-AMI acute abdominal pain [20]. Therefore, citrulline may be more useful in chronic or postoperative intestinal failure contexts rather than in emergency AMI diagnosis [1].

Ischemia-Modified Albumin

Ischemia-modified albumin (IMA) reflects structural alteration of albumin during oxidative stress and ischemia, reducing its metal-binding capacity. It has been studied in myocardial ischemia and has also been explored in AMI [6,24]. Early studies suggest potential diagnostic value, but the evidence base is limited, heterogeneous, and affected by the non-specific nature of systemic ischemia and oxidative stress [3,6,24]. IMA may rise in many acute illnesses, including sepsis, trauma, pulmonary embolism, renal disease, and cardiovascular events. Therefore, despite promising preliminary results, it cannot currently be recommended as a definitive diagnostic marker for AMI [1,2].

Alpha-Glutathione S-Transferase

Alpha-glutathione S-transferase (α-GST) is present in intestinal and hepatic tissue and may be released during mucosal injury. Early studies suggested that α-GST could rise during intestinal ischemia, but subsequent evaluations were inconsistent [6,25,26]. A review by Acosta et al. noted that follow-up studies produced inferior sensitivity and accuracy compared with preliminary reports, questioning their clinical value. In addition, hepatic injury can also elevate α-GST, reducing specificity in critically ill patients [25]. At present, α-GST remains investigational.

Procalcitonin and Interleukin-6

Procalcitonin (PCT) and interleukin-6 (IL-6) reflect SIR, bacterial translocation, and sepsis. Their diagnostic appeal in AMI lies in the progression from ischemia to mucosal barrier breakdown, bacterial translocation, and SIR [27,28]. A review of PCT in intestinal ischemia suggested moderate sensitivity and negative predictive value, but available studies included heterogeneous populations and often focused on advanced ischemia or experimental models [27]. PCT is better regarded as a marker of infectious or necrotic complications than as an early diagnostic marker [1].

IL-6 may rise early in inflammatory cascades and has been studied as part of biomarker panels. However, like PCT, it is non-specific and not routinely used for emergency AMI diagnosis [3,5]. These markers may help identify severe disease, transmural necrosis, or postoperative intestinal ischemia, but their independent diagnostic value remains uncertain.

MicroRNA

Recent experimental work has explored circulating microRNAs, metabolomic signatures, and endothelial injury markers such as pro-adrenomedullin. Although preliminary findings are encouraging, evidence remains limited to small observational studies, and none are ready for clinical implementation [29].

Multimarker strategies

Because AMI is pathophysiologically complex, a single biomarker may be unrealistic. A rational panel should combine markers of coagulation activation, enterocyte injury, systemic hypoperfusion, inflammation, and necrosis. For example, D-dimer may capture thrombotic activity, I-FABP may capture mucosal injury, lactate may capture systemic hypoperfusion, and PCT or IL-6 may capture inflammatory progression. However, reviews emphasize that combinations have rarely been studied prospectively and that existing evidence is too heterogeneous to support a validated biomarker algorithm [3-5,29].

Future biomarker studies should distinguish AMI subtypes, including embolic occlusion, arterial thrombosis, non-occlusive mesenteric ischemia, and MVT. They should also differentiate reversible ischemia from transmural necrosis, because the clinical decisions differ. The most useful biomarker may not be one that simply detects AMI, but one that identifies patients who need immediate revascularization, laparotomy, bowel resection, or second-look surgery [1-3,21,25]. Artificial intelligence (AI) and machine learning algorithms integrating laboratory variables, imaging findings, and clinical characteristics may further improve future diagnostic pathways [3,5].

Practical clinical interpretation

The practical message for clinicians is that laboratory biomarkers should support, not replace, clinical judgment and CTA. In a patient with sudden severe abdominal pain, atrial fibrillation, recent myocardial infarction, diffuse atherosclerosis, hypotension, vasopressor use, heart failure, thrombophilia, or unexplained metabolic deterioration, AMI should remain high on the differential, even if routine laboratory values are initially normal [1,2,21]. Normal lactate, normal pH, or absence of leukocytosis should not delay imaging [1,2,9,13].

Conversely, abnormal laboratory markers should be interpreted in context. High lactate, acidosis, leukocytosis, elevated CRP, elevated D-dimer, renal dysfunction, and rising PCT in a patient with abdominal pain or unexplained shock should prompt urgent CTA and surgical or vascular consultation. These findings may indicate advanced ischemia and should accelerate definitive diagnostic imaging and treatment [1,2,9,13,15,21,27].

Future directions

The future of laboratory diagnosis in AMI likely lies in prospective, multicenter studies that use standardized definitions, precise timing from symptom onset, uniform CTA criteria, operative or pathological confirmation when available, and clinically meaningful endpoints. Biomarker studies should include emergency department patients with undifferentiated abdominal pain, intensive care patients at risk for non-occlusive ischemia, and postoperative patients, in whom diagnosis is particularly difficult. Serial measurement may be more informative than a single value, especially for markers with short half-lives or dynamic release patterns. Integration with clinical prediction tools, AI-based imaging interpretation, and rapid point-of-care assays may improve diagnostic pathways. However, any biomarker algorithm must be tested against the current standard of urgent CTA and must prove that it reduces time to diagnosis, bowel necrosis, unnecessary laparotomy, mortality, or resource use [1-4].

Discussion

The ideal laboratory biomarker for AMI remains elusive. Despite more than three decades of investigation, no laboratory biomarker has demonstrated sufficient diagnostic accuracy to replace CTA in the diagnosis of AMI. The principal reason is that most currently available biomarkers reflect the downstream consequences of intestinal ischemia, including inflammation, coagulation activation, bacterial translocation, or tissue necrosis, rather than the ischemic insult itself. Consequently, many routinely available laboratory tests become abnormal only after clinically significant bowel injury has already occurred, limiting their value for early diagnosis [1-6,21,25]. The routine, emerging, and investigational biomarkers that have been explored in the diagnosis of AMI are summarized in Table 1.

**Table 1:** Summary of laboratory biomarkers investigated for the diagnosis of acute mesenteric ischemia AMI: acute mesenteric ischemia, CTA: computed tomography angiography, SIR: systemic inflammatory response

Biomarker	Biological rationale	Routine availability	Diagnostic value	Major limitations	Current clinical role
White blood cell count	Reflects SIR to intestinal ischemia and necrosis	Widely available	Frequently elevated, but non-specific	Elevated in virtually all inflammatory and infectious abdominal conditions; may be normal early	Supportive marker only; cannot confirm or exclude AMI
Neutrophil-to-lymphocyte ratio	Composite marker of SIR	Widely available	Some studies suggest an association with AMI and mortality	Nonspecific; influenced by infection, malignancy, corticosteroids, trauma	Supportive prognostic marker only
C-reactive protein	Acute-phase reactant produced in response to inflammation	Widely available	May correlate with disease severity and bowel necrosis	Delayed rise; poor specificity; normal in early disease	Adjunctive marker of inflammation, rather than diagnosis
Serum L-lactate	Indicates anaerobic metabolism and tissue hypoperfusion	Widely available	Associated with advanced ischemia, bowel necrosis, and a worse prognosis	Often normal during early ischemia; elevated in numerous non-ischemic conditions (shock, sepsis, liver dysfunction)	Prognostic marker and indicator of disease severity; normal values do not exclude AMI
Metabolic acidosis	Reflects systemic consequences of prolonged tissue hypoxia	Widely available	Suggests advanced ischemia	Late finding; highly non-specific	Supports suspicion in critically ill patients, but has limited diagnostic value
D-dimer	Indicates activation of coagulation and fibrinolysis during arterial or venous thrombosis	Widely available	High sensitivity reported in many studies; useful for increasing suspicion	Poor specificity; elevated in older age, infection, cancer, surgery, trauma, venous thromboembolism, and many cardiovascular disorders	Adjunctive "rule-out" marker in selected patients; should never replace CTA
Amylase	Released during intestinal injury or associated pancreatic hypoperfusion	Widely available	Occasionally elevated	Very poor specificity; may mimic pancreatitis	Limited clinical utility
Serum phosphate	Released from necrotic cells during advanced bowel injury	Widely available	May increase in transmural necrosis	Inconsistent findings; affected by renal dysfunction and acid-base disturbances	Not recommended for routine diagnosis
Procalcitonin	Reflects bacterial translocation and SIR	Widely available in many hospitals	May correlate with bowel necrosis and severe disease	Better marker of sepsis than early ischemia; insufficient sensitivity for early diagnosis	Useful for severity assessment, rather than diagnosis
Intestinal fatty acid-binding protein	Released rapidly from damaged mature enterocytes during mucosal ischemia	Limited availability	One of the most promising enterocyte-specific biomarkers; moderate-to-good sensitivity and specificity in some studies	Considerable heterogeneity between assays and studies; inconsistent validation; not routinely available	Investigational; potentially useful in combination with other biomarkers
D-lactate	Produced by intestinal bacteria; rises following mucosal barrier disruption	Limited availability	Theoretical early marker of intestinal injury	Variable diagnostic performance; not routinely measured	Investigational
Citrulline	Produced by functional small-intestinal enterocytes; decreases with enterocyte loss	Limited availability	Reflects enterocyte mass, rather than acute ischemia	Influenced by renal function and chronic intestinal disease; poor acute diagnostic performance	Not recommended for routine diagnosis
Ischemia-modified albumin	Oxidative stress alters albumin binding capacity during ischemia	Limited availability	Preliminary studies reported moderate diagnostic accuracy	Elevated in numerous ischemic and inflammatory disorders	Investigational
Alpha-glutathione S-transferase	Cytosolic enzyme released from injured intestinal epithelial cells	Limited availability	Early studies were promising	Subsequent studies failed to confirm adequate diagnostic accuracy	Investigational
Interleukin-6	Early pro-inflammatory cytokine released during intestinal injury	Limited availability	May correlate with disease severity	Poor specificity; influenced by systemic inflammation	Investigational

This challenge is compounded by the heterogeneous nature of AMI. Arterial embolism, arterial thrombosis, non-occlusive mesenteric ischemia, and MVT differ considerably in their pathophysiology, disease progression, and extent of bowel injury, resulting in substantial variability in biomarker release. Furthermore, the diagnostic performance of many biomarkers is influenced by the timing of blood sampling, while their specificity is reduced by common comorbidities, such as sepsis, shock, malignancy, renal dysfunction, and recent surgery. Methodological heterogeneity among published studies, including differences in assay techniques, diagnostic thresholds, reference standards, and patient selection, has further limited direct comparisons, and prevented the establishment of universally accepted cut-off values [3-6,20,29].

Among routinely available laboratory tests, serum lactate and D-dimer have received the greatest clinical attention. Lactate remains an important marker of disease severity, and is associated with transmural bowel necrosis and poor prognosis; however, normal lactate concentrations do not exclude early AMI, whereas elevated levels are encountered in numerous conditions associated with systemic hypoperfusion or impaired lactate clearance. Similarly, D-dimer demonstrates relatively high sensitivity in several studies, and may increase suspicion for thromboembolic forms of AMI, but its poor specificity substantially limits its usefulness as a standalone diagnostic test. Accordingly, neither marker should be interpreted in isolation or used to determine whether CTA should be performed [1,2,9,13-16].

Enterocyte-specific biomarkers, particularly I-FABP, remain the most promising candidates for future clinical application, because they directly reflect intestinal epithelial injury, rather than its systemic consequences. Nevertheless, published studies have reported inconsistent diagnostic performance, largely because of variations in assay methodology, timing of sample collection, patient populations, and disease stage. Similar limitations apply to D-lactate, citrulline, IMA, α-GST, and inflammatory cytokines, all of which remain investigational, despite encouraging preliminary findings [19-28].

Collectively, the available evidence suggests that the future of laboratory diagnosis is unlikely to rely on a single biomarker. Instead, multimarker strategies integrating indicators of enterocyte injury, coagulation activation, inflammation, and tissue hypoperfusion may prove more informative than any individual laboratory test [3,5,29]. Future prospective multicenter studies using standardized assays, predefined diagnostic thresholds, and uniform imaging or operative reference standards are needed to determine whether such biomarker panels can improve diagnostic accuracy, facilitate earlier diagnosis, and ultimately reduce bowel necrosis and mortality.

Based on the current evidence, laboratory investigations should be regarded as complementary, rather than definitive diagnostic tools. Their greatest value lies in increasing clinical suspicion, supporting risk stratification, and reinforcing the need for urgent CTA in patients with a compatible clinical presentation. Consistent with contemporary international guidelines, CTA remains the diagnostic cornerstone of AMI, and neither normal, nor abnormal laboratory findings should delay appropriate imaging or definitive treatment [1,2].

## Conclusions

No currently available laboratory marker can independently confirm or exclude AMI. Lactate, acidosis, leukocytosis, CRP, D-dimer, PCT, and other routine markers may increase suspicion or indicate advanced disease, but they lack adequate specificity and may be normal early. D-dimer appears sensitive but poorly specific. I-FABP, D-lactate, citrulline, ischemia-modified albumin, alpha-GST, IL-6, and related investigational biomarkers are biologically plausible but insufficiently validated for routine use. The diagnosis of AMI remains primarily clinical and radiological. Laboratory tests should be used as adjuncts that support early suspicion and urgent CTA, never as gatekeepers that delay definitive imaging or treatment.

## References

[1] Bala M, Catena F, Kashuk J (2022). Acute mesenteric ischemia: updated guidelines of the World Society of Emergency Surgery. World J Emerg Surg.

[2] Koelemay MJ, Geelkerken RH, Kärkkäinen J (2025). Editor's Choice - European Society for Vascular Surgery (ESVS) 2025 Clinical Practice Guidelines on the Management of Diseases of the Mesenteric and Renal Arteries and Veins. Eur J Vasc Endovasc Surg.

[3] Reintam Blaser A, Starkopf J, Björck M (2023). Diagnostic accuracy of biomarkers to detect acute mesenteric ischaemia in adult patients: a systematic review and meta-analysis. World J Emerg Surg.

[4] Blauw JT, Metz FM, Nuzzo A (2024). The diagnostic value of biomarkers in acute mesenteric ischaemia is insufficiently substantiated: a systematic review. Eur J Vasc Endovasc Surg.

[5] Zafirovski A, Zafirovska M, Kuhelj D, Pintar T (2023). The impact of biomarkers on the early detection of acute mesenteric ischemia. Biomedicines.

[6] Peoc'h K, Nuzzo A, Guedj K, Paugam C, Corcos O (2018). Diagnosis biomarkers in acute intestinal ischemic injury: so close, yet so far. Clin Chem Lab Med.

[7] Acosta S (2015). Mesenteric ischemia. Curr Opin Crit Care.

[8] Kanda T, Fujii H, Tani T (1996). Intestinal fatty acid-binding protein is a useful diagnostic marker for mesenteric infarction in humans. Gastroenterology.

[9] Isfordink CJ, Dekker D, Monkelbaan JF (2018). Clinical value of serum lactate measurement in diagnosing acute mesenteric ischaemia. Neth J Med.

[10] Kurt Y, Akin ML, Demirbas S, Uluutku AH, Gulderen M, Avsar K, Celenk T (2005). D-dimer in the early diagnosis of acute mesenteric ischemia secondary to arterial occlusion in rats. Eur Surg Res.

[11] Aktimur R, Cetinkunar S, Yildirim K, Aktimur SH, Ugurlucan M, Ozlem N (2016). Neutrophil-to-lymphocyte ratio as a diagnostic biomarker for the diagnosis of acute mesenteric ischemia. Eur J Trauma Emerg Surg.

[12] Tanrıkulu Y, Şen Tanrıkulu C, Sabuncuoğlu MZ, Temiz A, Köktürk F, Yalçın B (2016). Diagnostic utility of the neutrophil-lymphocyte ratio in patients with acute mesenteric ischemia: a retrospective cohort study. Ulus Travma Acil Cerrahi Derg.

[13] Collange O, Lopez M, Lejay A (2022). Serum lactate and acute mesenteric ischaemia: an observational, controlled multicentre study. Anaesth Crit Care Pain Med.

[14] Studer P, Vaucher A, Candinas D, Schnüriger B (2015). The value of serial serum lactate measurements in predicting the extent of ischemic bowel and outcome of patients suffering acute mesenteric ischemia. J Gastrointest Surg.

[15] Sun DL, Li SM, Cen YY (2017). Accuracy of using serum D-dimer for diagnosis of acute intestinal ischemia: a meta-analysis. Medicine (Baltimore).

[16] Acosta S, Nilsson TK, Björck M (2004). D-dimer testing in patients with suspected acute thromboembolic occlusion of the superior mesenteric artery. Br J Surg.

[17] Karabulut K, Gül M, Dündar ZD, Cander B, Kurban S, Toy H (2011). Diagnostic and prognostic value of procalcitonin and phosphorus in acute mesenteric ischemia. Ulus Travma Acil Cerrahi Derg.

[18] Hot S, Egin S, Ilhan M (2021). The value of potassium, pH and D-dimer levels in early diagnosis of acute mesenteric ischemia: an experimental study on rats. Arch Med Sci.

[19] Sun DL, Cen YY, Li SM, Li WM, Lu QP, Xu PY (2016). Accuracy of the serum intestinal fatty-acid-binding protein for diagnosis of acute intestinal ischemia: a meta-analysis. Sci Rep.

[20] Nuzzo A, Guedj K, Curac S (2021). Accuracy of citrulline, I-FABP and D-lactate in the diagnosis of acute mesenteric ischemia. Sci Rep.

[21] Nuzzo A, Maggiori L, Ronot M (2017). Predictive factors of intestinal necrosis in acute mesenteric ischemia: prospective study from an intestinal stroke center. Am J Gastroenterol.

[22] Salim SY, Young PY, Churchill TA, Khadaroo RG (2017). Urine intestinal fatty acid-binding protein predicts acute mesenteric ischemia in patients. J Surg Res.

[23] Shi H, Wu B, Wan J, Liu W, Su B (2015). The role of serum intestinal fatty acid binding protein levels and D-lactate levels in the diagnosis of acute intestinal ischemia. Clin Res Hepatol Gastroenterol.

[24] Gunduz A, Turedi S, Mentese A (2008). Ischemia-modified albumin in the diagnosis of acute mesenteric ischemia: a preliminary study. Am J Emerg Med.

[25] Acosta S, Nilsson T (2012). Current status on plasma biomarkers for acute mesenteric ischemia. J Thromb Thrombolysis.

[26] Kazmi SS, Safi N, Berge ST (2022). Plasma α-glutathione S-transferase in patients with chronic mesenteric ischemia and median arcuate ligament syndrome. Vasc Health Risk Manag.

[27] Cosse C, Sabbagh C, Kamel S, Galmiche A, Regimbeau JM (2014). Procalcitonin and intestinal ischemia: a review of the literature. World J Gastroenterol.

[28] Karaca Y, Gündüz A, Türkmen S, Menteşe A, Türedi S, Eryiğit U, Karahan SC (2015). Diagnostic value of procalcitonin levels in acute mesenteric ischemia. Balkan Med J.

[29] Memet O, Zhang L, Shen J (2019). Serological biomarkers for acute mesenteric ischemia. Ann Transl Med.

